# Core–Shell CoS_2_/FeS_2_ Heterojunction Encapsulated in N-Doped Carbon Nanocubes Derived from Coordination Polymers for Electrocatalytic Alkaline Water/Seawater Splitting

**DOI:** 10.3390/polym17121701

**Published:** 2025-06-19

**Authors:** Xiaoyin Zhang, Yan Liu, Zihan Zeng, Yan Zou, Wanzhen Wang, Jing Zhang, Jing Wang, Xiangfeng Kong, Xiangmin Meng

**Affiliations:** 1 Institute of Oceanographic Instrumentation, Qilu University of Technology (Shandong Academy of Sciences), Qingdao 266061, China; zhangxiaoyin301@163.com (X.Z.);; 2 College of Marine Science and Biological Engineering, Qingdao University of Science and Technology, Qingdao 266042, Chinamengxiangmin66@163.com (X.M.); 3 Modern Industrial College of Biomedicine and Great Health, Youjiang Medical University for Nationalities, Baise 533000, China

**Keywords:** heterojunction, coordination polymers, core–shell, electrocatalysis, seawater splitting

## Abstract

Utilizing renewable energy for green hydrogen production via electrolyzed seawater is a promising technology for the future. However, undesired chlorine evolution and the corrosive nature of seawater are crucial challenges for direct seawater splitting technology. In this work, heterojunctions of CoS_2_/FeS_2_ encapsulated in N-doped carbon nanocubes (denoted as CoS_2_/FeS_2_@NC) were designed by proposing the synchronous pyrolysis and vulcanization of polydopamine-coated coordination polymers. Such a synthetic strategy was demonstrated to be effective in increasing the favorable exposure of active sites, moderately regulating electronic structure, and remarkably facilitating charge transfer due to the controllable generation of unique core–shell structures with suitable carbon shells, leading to the excellent bifunctional electrocatalytic performance and enhanced stability of electrocatalysts. As a result, CoS_2_/FeS_2_@NC can be revealed as a superior water splitting catalyst, possessing a small voltage of 1.75 V and requiring 100.0 mA cm^−2^ in 1 M KOH alkaline solution and 1.80 V for alkaline seawater media, with satisfactory long-term stability. This work presents fresh strategies for designing core–shell heterostructures and developing green technology for hydrogen production.

## 1. Introduction

Hydrogen, as an environmentally friendly energy carrier, has been considered as the most promising candidate as a form of energy to replace traditional fossil fuels due to its high energy density, near-zero carbon pollution, and recyclable combustion products [1,2,3,4]. Among different hydrogen production methods, electrochemical water splitting has become the most practical technology for hydrogen production because of its ability to achieve the sustainable production of green hydrogen [5,6,7]. At present, there are two urgent problems to be solved in the electrolytic hydrogen production system: (1) Almost all electrolytic water systems require fresh water resources as electrolytes, and the large-scale electrolysis of fresh water not only exacerbates the problem of water scarcity, but also involves expensive water purification systems; (2) the slow kinetics of chemical reactions and complex electron transfer involved in the electrolysis process result in high overpotential and energy consumption, greatly limiting the overall electrolysis efficiency by leading to increased electrolysis costs [8,9,10].

Seawater, as a natural multi-component salt solution, is abundant in natural resources and has good ionic conductivity [11]; thus, using seawater instead of fresh water for electrolytic hydrogen production is highly advantageous. More importantly, combined with renewable marine energy such as offshore solar energy, wind energy, and tidal energy, direct seawater electrolysis (DSE) can further improve the utilization of marine resources, which enables the promoted application of electrocatalytic hydrogen production in fresh water-scarce areas [12,13,14]. Nevertheless, the chemical complexity of seawater presents substantial challenges such as electrode corrosion, chlorine ion competition oxidation, and electrolytic cell failure, causing intractable problems in the technology of DSE in terms of economic feasibility [15,16,17,18]. In this regard, improving the selectivity and stability of electrocatalysts is an effective way to enhance the overall efficiency of seawater electrolysis for hydrogen production.

Nowadays, the commercial HER and OER electrocatalysts are Pt-group metals (such as Pt, Ru, Ir) or their derivative compounds [19,20,21]. However, the scarce resources, expensive price, and insufficient electrochemical stability seriously hinder the industrial development of electrocatalytic hydrogen production. Transition metal sulfides (TMSs), particularly Fe/Co-based sulfides, have garnered increasing attention owing to their excellent conductivity and high catalytic activity, due to which the strong synergistic effect between the active sites could regulate the distribution of electron density, thereby effectively enhancing the electrocatalytic activity [22,23,24]. However, the unsatisfactory electrocatalytic activity and long term-stability caused by the low conductivity and easy aggregation of Co/Fe-based materials made them unable to completely substitute commercial Pt-group electrocatalysts. Currently, integrating conductive carbon to form composite materials has been considered as an effective strategy to enhance the electrochemical activity and durability of metal sulfide catalysts [25,26]. Coordination polymers (CPs), with the advantages of large specific surface area, uniform dispersion of catalytic active sites, and controllable pore size, have received increasing attention as ideal carriers for the preparation of carbon-supported Co/Fe-based catalysts [27,28,29], which contain large numbers of organic ligands that can be originally generated as carbon matrixes of electrocatalysts during the pyrolysis process. 

Based on the above considerations, we reported the construction of a core–shell CoS_2_/FeS_2_ heterojunction encapsulated in N-doped carbon nanocubes as a bifunctional electrocatalyst for alkaline water/seawater splitting by in situ pyrolysis of the precursor polydopamine-coated CPs. Such a unique N-doped carbon matrix was formed from a CoFe-CPs precursor, which not only protected the CoS_2_/FeS_2_ heterojunction from aggregation during the electrocatalysis process, but also assisted in the better conductivity and increased specific surface area of the as-prepared electrocatalysts. Consequently, the as-prepared samples of CoS_2_/FeS_2_@NC demonstrated excellent electrocatalytic activity for overall water and seawater splitting with relatively low cell voltages of 1.67 V and 1.80 V for delivering the current density of 100 mA cm^−2^ in alkaline media, respectively, which were favorably comparable to the commercial Pt/C and IrO_2_ benchmarks.

## 2. Materials and Methods

### 2.1. Materials

All chemicals, including cobalt (II) nitrate hexahydrate (CoNO_3_·6H_2_O, AR), potassium hexacyanoferrate (III) (K_3_[Fe(CN)_6_], AR), trisodium citrate dihydrate (Na_3_C_6_H_5_O_7_·2H_2_O, AR), dopamine (AR), sublimed sulfur (S, AR), ethanol (AR), and Nafon (5 wt %), were purchased from Sigma-Aldrich (Shanghai, China) and used as received without any purification. Deionized water (DI, 18 MΩ) used for all experiments was supplied by a Millipore system (Millipore, MA, USA).

### 2.2. Synthesis of Co-Fe Hybrid Coordination Polymer (CoFe-CPs)

CoFe-CPs was synthesized through a coprecipitation method, CoNO_3_·6H_2_O (12 mmol, 3.49 g) and Na_3_C_6_H_5_O_7_·2H_2_O (15 mmol, 4.42 g) were dissolved in 200 mL of DI water to form solution A. K_3_[Fe(CN)_6_] (8 mmol, 2.63 g) was dissolved into 100 mL of DI water to form solution B. Then, solutions A and B were thoroughly mixed under magnetic stirring for 2 h, followed by 22 h aging. The obtained precipitate solid was collected by centrifugation, washed with DI water and ethanol for several times, and then dried overnight at 70 °C in an oven.

### 2.3. Synthesis of Polydopamine Coated CoNi-CPs Hollow Cubes (CoFe-CPs@PDA)

In total, 0.2 g of CoFe-CPs nanocubes were dispersed into 100 mL Tris-buffer solution (pH = 8.5, 10 mM) with ultrasonication for 20 min, and then 0.04 g dopamine was added into the solution, followed by stirring for 24 h. The resultant was collected via centrifugation and washed with deionized water and ethanol for several times, respectively. The final product was dried under vacuum at 50 °C overnight. In addition, the other four samples with different additions of dopamine (0.02 g, 0.05 g, 0.1 g and 0.2 g) were prepared to investigate the effect of PDA loading amounts.

### 2.4. Synthesis of CoS_2_/FeS_2_@NC Nanocubes

In a typical procedure, the porcelain boat with the prepared CoFe-CPs@PDA precursor (100 mg) was placed at the center of the tube furnace, with 300 mg of sulfur powder placed at the upstream side of the furnace as the S source. Impurity gas in the furnace was purged with Ar gas (purity, 99.999%) for 30 min. Afterward, the furnace was the tube was heated to 400 °C with a rate of 2 °C min^−1^ under the flowing Ar atmosphere and kept at this temperature for 3 h. Finally, the furnace was naturally cooled down to room temperature with Ar gas flowing. The confined biactive CoS_2_/FeS_2_ in N-doped carbon nanocubes (CoS_2_/FeS_2_@NC) was obtained. The effect of pyrolysis temperatures (300 and 500 °C) was also explored, and the products were denoted as CoS_2_/FeS_2_@NC-300 and CoS_2_/FeS_2_@NC-500, respectively. Additionally, CoS_2_/FeS_2_ was prepared using the same condition with CoS_2_ /FeS_2_@NC, with the exception that CoFe-CPs@PDA was replaced with CoFe-CPs.

### 2.5. Characterization

The morphologies of the as-prepared samples were analyzed by a field emission scanning electron microscope (SEM, S-4800, Hitachi, Japan), transmission electron microscopy (TEM, Tecnai G2-F20, Thermo Fisher Scientific, Waltham, MA, USA), high-resolution transmission electron microscopy (HR-TEM, JEM-2100F, JEOL, Tokyo, Japan), and a high-angle annular dark-field scanning transmission electron microscope (HAADF-STEM, JEM-ARM200F, JEOL, Japan). The crystal structure was examined by X-ray diffraction (XRD, D8-Advance diffractometer, Bruker, Bremen, Germany) using Cu Kα radiation. The valence states were detected by X-ray photoelectron spectroscopy (XPS, AXISULTRA DLD) on a Phi X-tool XPS instrument (Shimadzu, Kyoto, Japan) with an Al Kα X-ray source. Raman spectra were discerned via a laser Raman spectrometer (Renishaw in Via plus, Renishaw, Sheffield, UK) with an excitation laser of 514 nm. The obtained adsorption–desorption isotherms were evaluated using the Micromeritics Tristar II 3020M (Micromeritics, Norcross, GA, USA) to provide the pore parameters including Brunauer–Emmett–Teller (BET) specific surface area and pore size. The pore size distribution was calculated using the HK method. 

### 2.6. Electrocatalytic Measurements 

In a typical prepared procedure of the working electrode, 50 µL of the homogeneous ink, which was prepared by dispersing 8 mg sample and 80 µL Nafion solution (5 wt%) in 1920 µL ethanol solution, was loaded onto the two sides of the carbon fiber paper (CFP) electrode (0.5 × 1 cm^2^) with the desired loading mass of 0.4 mg/cm^2^. Prior to use, the CFP (FuelCell Store) was treated in a mixed solution of sulfuric acid and nitric acid (v, 98% H_2_SO_4_): v (70%, HNO_3_): v(H_2_O) = 1:1:1 at 60 °C for 24 h under vigorous magnetic stirring. 

All electrochemical measurements were conducted at room temperature (≈25 °C) in a typical three-electrode or two-electrode configuration using the CHI 760 E Electrochemical Workstation (CHI Instruments, Shanghai Chenhua Instrument Corp., Shanghai, China). The HER performance was evaluated in alkaline seawater (pH = 14) or 1.0 M KOH (pH = 14) solution with as-fabricated CFP as the working electrode, Ag/AgCl as the reference electrode, and graphite rod as the counter electrode. The alkaline seawater was from Jiaozhou Bay in Qingdao, China.

The OER performance was evaluated with the same three-electrode configuration in 1.0 M KOH solution or alkaline seawater, but a Pt plate was used as the counter electrode. The full electrolyzer cell was assembled using two identical CFP electrodes with as-fabricated CoS_2_ /FeS_2_@NC and measured in a two-electrode mode. In this report, all potentials were scaled with respect to the RHE by the following equation: E*_RHE_* = E_Ag/AgCl_ + 0.197 + 0.059 × pH. The linear sweep voltammetry (LSV) curves were collected with a scan rate of 5 mV·s^−1^. Electrochemical impedance spectroscopy (EIS) was tested at a constant potential of 1.60 V (vs. RHE) from 10,000 to 0.1 Hz with an AC potential magnitude of 10 mV. The electrical double-layer capacitor (C_dl_) values were obtained from CV plots in a non-Faradaic small window of 0.06–0.18 V (vs. RHE). The electrode durability was conducted at 10 mA·cm^−2^ for 36,000 s by using chronoamperometry.

## 3. Results and Discussion

### 3.1. Textural and Chemical Characterization

**Scheme 1:**
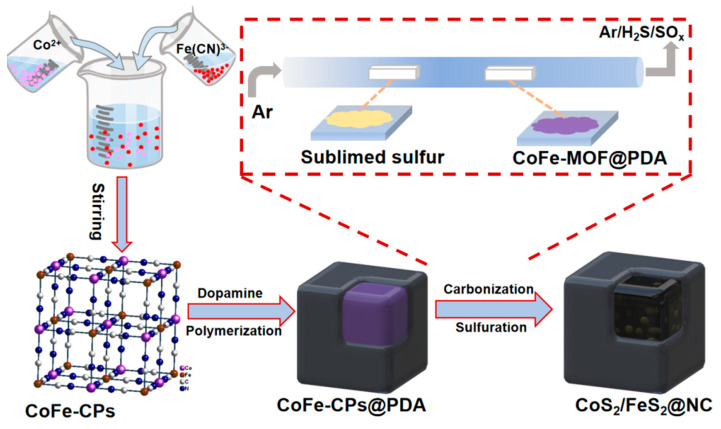
Schematic illustration of the preparation of CoS_2_/FeS_2_@NC.

The synthetic procedure of CoS_2_/FeS_2_@NC core–shell nanocubes is graphically depicted in Scheme 1. Firstly, the uniform CoFe-CPs nanocubes were obtained by the facile chemical precipitation of Co^2+^ cations and Fe(CN)_6_^3−^ anions in the presence of sodium citrate under room temperature. Secondly, the as-prepared CoFe-CPs were coated with polydopamine (PDA) to form core–shell CoFe-MOFs@PDA structures, where the PDA was produced by self-polymerization of dopamine in the alkaline buffer solution and uniformly covered on the surface of CoFe-CPs nanocubes. Finally, the CoFe-CPs@PDA precursor was transformed into CoS_2_/FeS_2_ hetero-nanocrystals encapsulated into N-doped carbon core–shell nanocubes (CoS_2_/FeS_2_@NC) after high-temperature carbonization (400 °C) and a sulphuration reaction in Ar atmosphere. 

**Figure 1:**
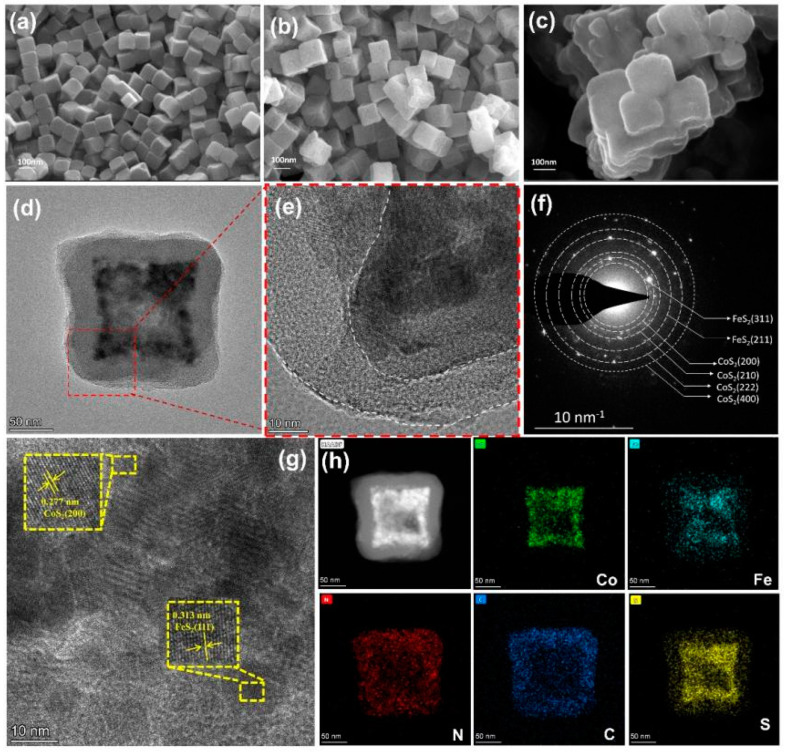
(**a**) SEM images of CoFe-CPs, (**b**) CoFe-CPs@PDA, and (**c**) CoS_2_/FeS_2_@NC; (**d**,**e**) TEM images and (**f**) the corresponding SAED pattern of CoS_2_/FeS_2_@NC; (**g**) HRTEM image and the lattice spacing corresponding to the selected areas in panel of CoS_2_/FeS_2_@NC; (**h**) the HAADF-TEM image and the corresponding element mapping of CoS_2_/FeS_2_@NC.

Figure 1a and Figure S1 show the morphology of the CoFe-CPs nanocubes with the average size of 100 nm. After the polydopamine was coated, the size of these nanocubes increased slightly and the surface became rough (Figure 1b and Figure S1), and the final products after the synchronous carbonization and sulphuration and the morphology of sample completely retained the typical cubical shape (Figure 1b). The Brunauer–Emmett–Teller (BET) surface area of (CoS_2_/FeS_2_@NC) was obtained by N_2_ adsorption/desorption isotherm as 202.5137 m^2^/g; such a large surface area would provide high exposure of active sites for the improved performance of catalysts (Figure S2) [30]. In order to further investigate the architectural feature of each nanocube in the CoS_2_/FeS_2_@NC, transmission electron microscopy (TEM) measurement was carried out. As reflected in Figure 1d,e, the as-prepared samples displayed a typical core–shell structure, where the CoS_2_/FeS_2_ hetero-nanocrystals were uniformly wrapped in the N-doped carbon shell with a thickness of 15–30 nm, which could prevent the aggregation of active sites effectively and enhance the stability during the electrocatalytic processing [31]. The corresponding selected area electron diffraction (SAED) patterns of CoS_2_/FeS_2_@NC nanocubes were indexed to polycrystalline diffraction rings of CoS_2_ and FeS_2_, as marked in the Figure 1f. A high-resolution transmission electron microscopy (HR-TEM) image is shown in Figure 1g, where the identified d-spacing of 0.277 nm was attributed to (200) of CoS_2_, and the spacing of 0.313 nm corresponded to (111) of FeS_2_, consistent with the SAED pattern. In addition, the high-angle annular dark-field scanning transmission electron microscope (HAADF-TEM) image and the corresponding element mapping demonstrated that Fe, Co, N, S, and C were uniformly distributed throughout the nanocubes. 

**Figure 2:**
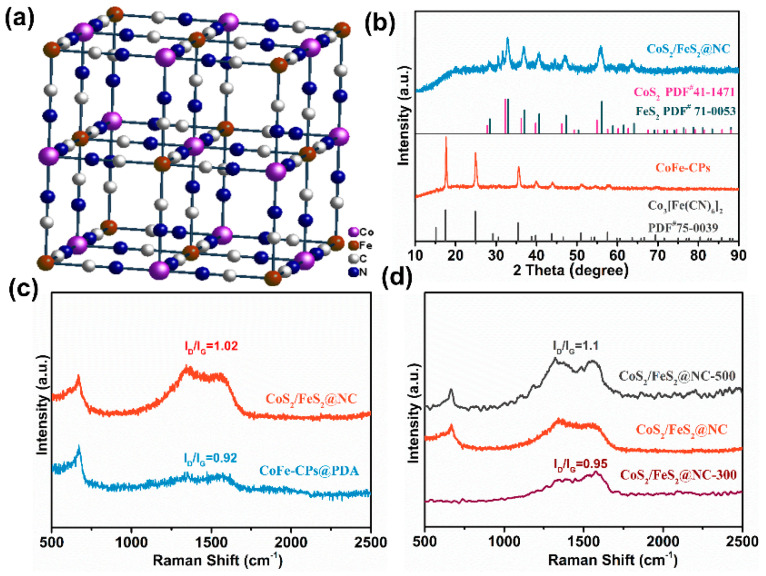
(**a**) The crystal structure of Co_3_[Fe(CN)_6_]_2_; (**b**) XRD patterns of CoFe-CPs and CoS_2_/FeS_2_@NC. (**c**) Raman spectra of CoFe-CPs@PDA and CoS2/FeS2@NC; (**d**) Raman spectra of the CoS_2_/FeS_2_@NC-300, CoS_2_/FeS_2_@NC, and CoS_2_/FeS_2_@NC-500.

The crystalline nature of the as-prepared samples was analyzed by powder X-ray diffraction (XRD). As shown in Figure 2a,b, the diffractogram of CoFe-MOFs could be effectively assigned to Co_3_[Fe(CN)_6_]_2_ (PDF#75-0039), and the characteristic peaks at 17.5°, 24.6°, and 35.1°could correspond to the (200), (220), and (400) crystallographic planes, respectively [32]. Moreover, as shown in Figure S3a, the diffraction pattern of the Co_3_[Fe(CN)_6_]_2_ nanocubes was in line with the cubic isostructure simulated from COD no.1010375 (space group: F-43m; *a* = *b* = *c* = 10.08 Å), and the intense characteristic peaks and pure crystalline phase manifested the good crystallinity and purity of the as-prepared CoFe-CPs sample [33]. For the final product of CoS_2_/FeS_2_@NC, the XRD pattern was consistent with the cubic cattierite structure of CoS_2_ with a space group of Pa-3 (PDF#41-01471; *a* = *b* = *c* = 5.5376 Å) and the cubic pyrite structure of FeS_2_ with a space group of Pa-3 (PDF#71-0053; *a* = *b* = *c* = 5.4281 Å), which confirmed the complete transformation from the precursor to the Co_2_S/FeS_2_@NC core–shell nanocubes (Figure 2b). Moreover, the XRD patterns of the control product CoS_2_/FeS_2_ are shown in Figure S3b, and the typical diffraction peaks of CoS_2_ as well as the characteristic peaks of FeS_2_ were obviously observed [34]. The structural change between CoFe-CPs@PDA and CoS_2_/FeS_2_@NC that happened during the carbonization and sulphuration was also investigated by Raman spectroscopy. As seen from Figure 2c, the Raman spectrum of CoS_2_/FeS_2_@NC displayed the D band peak (the vibrations of carbon atoms with sp^3^ electronic configuration) at ~1305 cm^−1^ and the G band peak (the vibration of sp^2^-hybridized carbon atoms) at ~1583 cm^−1^. The intensity ratio I_D_/I_G_ of CoS_2_/FeS_2_@NC was calculated as 1.02, which showed that the I_D_/I_G_ was higher than that of the precursor CoFe-CPs@PDA (0.92), suggesting the decrease in the average size of the sp^3^ domains after the carbonization process with a reduction in the coating PDA layer [35]. In addition, catalysts obtained from different pyrolysis temperatures were also compared by Raman spectra; in Figure 2d, it can be seen that the values of I_D_/I_G_ increased with the increasing the carbonization temperatures.

**Figure 3:**
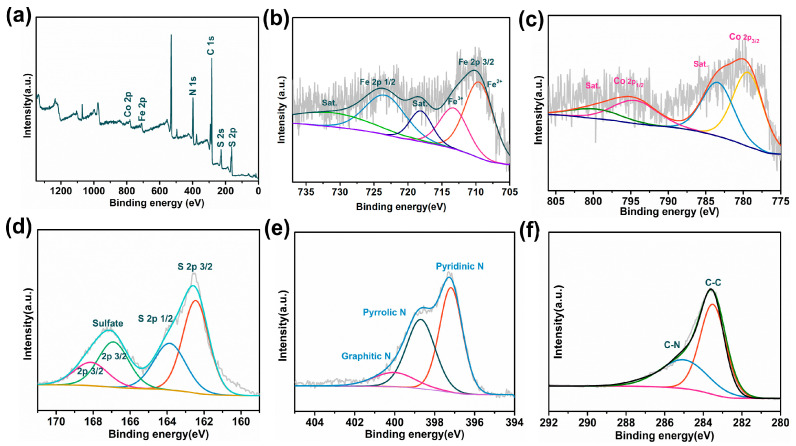
XPS spectra of obtained CoS_2_/FeS_2_@NC; (**a**) Survey spectrum, (**b**) Fe 2p, (**c**) Co 2p, (**d**) S2p, (**e**) N1s and (**f**) C1s.

X-ray photoelectron spectroscopy (XPS) measurement was carried out to evaluate the valence state and the surface chemical composition of CoS_2_/FeS_2_@NC. As shown in Figure 3a, the full survey spectra demonstrated the coexistence of Co, Fe, C, N, and S elements, corresponding to the results of the EDS test. The high-resolution Fe 2p spectra are shown in Figure 3b; two peaks at 708.6 and 714.7 eV separated from the Fe 2p3/2 can be attributable to +2 and +3 states, respectively. Binding energy at 721.90 eV corresponded to Fe 2p1/2 electronic configurations. The other two peaks at 719.1 eV and 732.9 eV could be assigned to the satellite peaks. The Co 2p spectrum in Figure 3c was separated into Co 2p 3/2 (778.9 eV), Co 2p 1/2 (794.3 eV), and satellite (784.4 eV and 799.1 eV) species, which corresponded to +2 states of Co [36]. The S2p spectra were divided into four peaks at 161.1 eV, 164.2 eV, 167.3 eV, and 168.1eV, attributed to 2p 2/3 and 2p1/2 splitting from the S2p spin orbital (-C-S-C) and sulfate species, respectively (Figure 3d). The N 1s spectra could be deconvoluted into three peaks at 401.0 eV, 400.1 eV, and 398.3 eV, demonstrating the coexistence of graphitic N, pyrrolic N, and pyridinic N in the CoS_2_/FeS_2_@NC (Figure 3e). In Figure 3f, the C1s spectrum displayed two peaks at 283.3 and 285.2 eV, corresponding to the C-C and C-N bonds, respectively.

### 3.2. OER Catalytic Activity of CoS_2_/FeS_2_@NC in Alkaline Water

Firstly, the effect of pyrolysis temperatures on the electrocatalytic performance was investigated. As Figure S4a displays, the catalysts obtained at 400 °C showed the best activity both for HER and OER in 1 M KOH solution. In accordance with results of Raman spectra, it can be concluded that the graphitization degree of the carbon shell in CoS_2_/FeS_2_@NC may be a factor affecting the electrocatalytic performance [37]. Moreover, the influence of carbon layer thickness has also been investigated by tuning the amount of dopamine during the coating process. Figure S4b shows that the optimized catalyst with the ratio of 1:5 (CoFe-CPs to dopamine) possessed the smallest overpotential and highest current density in the applied potential, demonstrating that the thickness of the carbon shell presented a noticeable effect on enhancing the electrocatalytic performance [38].

**Figure 4:**
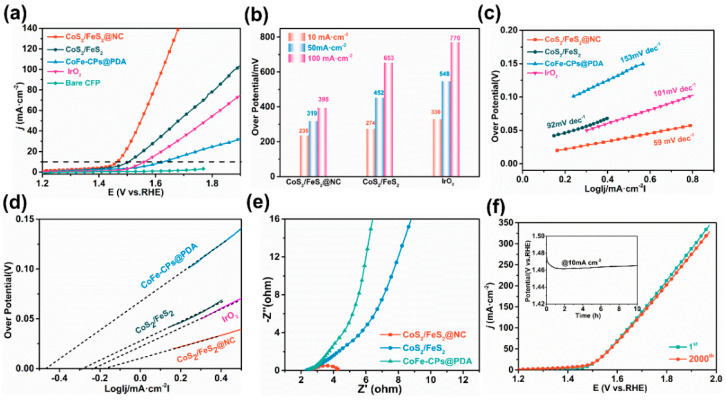
(**a**) LSV curves for OER in 1.0 M KOH solution; (**b**) comparation of overpotentials to deliver 10, 50, and 100 mA·cm^−2^; (**c**) Tafel plots; (**d**) exchange current density; (**e**) Nyquist plots applied at the onset potential; (**f**) LSV curves of CoS_2_/FeS_2_@NC in 1st and 2000th potential cycles (inset shows chronotentiometric curves at 10 mA·cm^−2^).

The Oxygen Evolution Reaction (OER) performance of as-prepared CoS_2_/FeS_2_@NC was investigated in 1.0 M KOH solution. For comparison, the electrocatalytic activity of CoS_2_/FeS_2_, CoFe-CPs@PDA, and commercially available IrO_2_ catalysts was also measured. The linear sweep voltammetry (LSV) curves in Figure 4a,b show that CoS_2_/FeS_2_@NC exhibited much higher OER performance with the overpotential (ηi = Ei − 1.23 V) of 235 mV to achieve the current density of 10 mA cm^−2^, lower than that of CoS_2_/FeS_2_ (274 mV) and CoFe-CPs@PDA (398 mV), as well as IrO_2_ (330 mV), which directly supported the concept that the synergistic effect between these two metals and the unique core–shell morphology could promote the catalytic performance. The Tafel slope of CoS_2_/FeS_2_@NC measured to be 59 mV·dec^−1^ was much smaller than that of CoS2/FeS2 (92 mV·dec^−1^), CoFe-CPs@PDA (153 mV·dec^−1^), and IrO_2_ (101 mV·dec^−1^) (Figure 4c). In addition, the Tafel slope was closely related to the inherent catalytic kinetics of the electrocatalyst, and the low Tafel slope of CoS2/FeS2@NC revealed the fast charge transfer and high efficiency of O_2_ generation during electrocatalysis processing. Moreover, exchange current density (j_0_) was also calculated from the corresponding Tafel slopes. As shown in Figure 4d, CoS_2_/FeS_2_@NC displayed the largest value of 0.21 mA·cm^−2^ compared to CoS_2_/FeS_2_ (0.3 mA·cm^−2^), CoFe-CPs@PDA (0.46 mA·cm^−2^), and IrO_2_ (0.25 mA·cm^−2^), showing the optimal OER kinetics of CoS_2_/FeS_2_@NC during catalytic processes. 

Electrochemical impedance spectroscopy (EIS) was applied to further research the reaction kinetics and charge transfer of the Co_S2_/FeS2@NC electrode. The semicircular radius of CoS_2_/FeS_2_@NC shown in Figure 4e was significantly lower than that of CoS_2_/FeS_2_ and CoFe-CPs@PDA, which may be due to the excellent conductivity of the N-doped carbon nanocubes’ structure which could effectively promote charge transfer and prevent aggregation to high-performance OER activity during electrochemical reactions.

The long-term operation of OER reactions is also critical in practical applications for water electrolysis and is one of the key parameters in the design of electrocatalysts. To study the stability of the CoS_2_/FeS_2_@NC electrode, an accelerated degradation test (ADT) was carried out via scanning cyclic voltammetry (CV). As displayed in Figure 4f, the polarization curve after 1000 continuous CV cycles almost overlapped with the original one. Moreover, the current density of CoS_2_/FeS_2_@NC kept steady after 24 h of i-t chronotentiometric measurement, demonstrating the excellent long-term durability of CoS_2_/FeS_2_@NC under the protection of N-doped carbon nanocubes.

In order to better understand the basic mechanism for the enhanced performance of CoS_2_/FeS_2_@NC, the electrochemical active surface areas (ECSAs) were explored by testing the electrochemical double layer capacitance (EDLC) at the solid–liquid interface electrodes. The EDLC was evaluated within a certain voltage range in the non-Faradaic region (Figure S6a–c). The Cdl value of CoS2/FeS_2_@NC (64 mF·cm^−2^) was greater than that of CoS_2_/FeS_2_ (39 mF·cm^−2^) and CoFe-CPs@PDA (33 mF·cm^−2^), demonstrating that the core–shell CoS_2_/FeS_2_@NC nanocomposites had the highest surface area and more exposure of active sites, which may be one of the main reasons for the enhanced electrocatalytic performance of CoS_2_/FeS_2_@NC [39] (Figure S6d).

### 3.3. HER Catalytic Activity of CoS_2_/FeS_2_@NC in Alkaline Water

**Figure 5:**
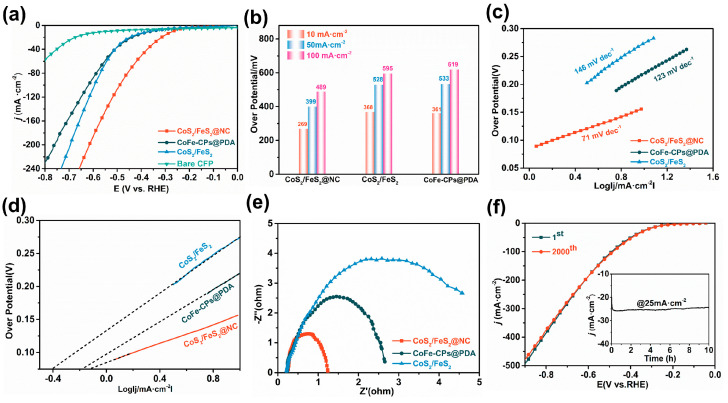
(**a**) LSV curves for HER in 1.0 M KOH solution; (**b**) comparation of overpotentials to deliver 10, 50, and 100 mA·cm^−2^; (**c**) Tafel plots; (**d**) exchange current density; (**e**) Nyquist plots at applied at the onset overpotential; (**f**) LSV curves of CoS_2_/FeS_2_@NC in 1st and 2000th potential cycles (inset shows i−t curves at −400 mV) of 100 mV.

The Hydrogen Evolution Reaction (HER) performances of CoS_2_/FeS_2_@NC were investigated in 1 M KOH solution and also compared with that of other prepared catalysts. As shown in Figure 5a,b, among all the as-synthesized catalysts, CoS_2_/FeS_2_@NC exhibits the highest catalytic activity with the overpotential of 269 mV at the current density of 10 mA cm^−2^, followed by CoS_2_/FeS_2_ and CoFe-CPs@PDA with the overpotential of 362 mV and 368 mV, respectively. The Tafel slope of CoS_2_/FeS_2_@NC was 71 mV·dec^−1^, which was much smaller relative to CoS_2_/FeS_2_ (146 mV·dec^−1^) and CoFe-CPs@PDA (123 mV·dec^−1^) (Figure 5c). The exchange current density (j_0_) calculated from the Tafel slopes is shown in Figure 5d; CoS_2_/FeS_2_@NC showed the largest value of 0.21 mA·cm^−2^ compared to CoS2/FeS2 (0.3 mA·cm^−2^) and CoFe-CPs@PDA (0.46 mA·cm^−2^). The Nyquist plots of as-prepared samples were further studied, and the semicircular radius of CoS_2_/FeS_2_@NC was smaller than that of CoS_2_/FeS_2_ and CoFe-CPs@PDA, demonstrating the lower charge-transfer resistance on the surface of CoS_2_/FeS_2_@NC during HER processing (Figure 5e). The stability of the CoS_2_/FeS_2_@NC electrode for HER was investigated by performing CV and i-t curves in 1 M KOH medium. As shown in Figure 5f, the CV cycles almost overlap with the initial cycle after 2000 continuous scanning cycles, and the current density could keep steady for 10 h, demonstrating the excellent long-term durability of the CoS_2_/FeS_2_@NC electrode for HER.

### 3.4. Electrocatalytic Activity of CoS_2_/FeS_2_@NC for Water/Seawater Splitting

**Figure 6:**
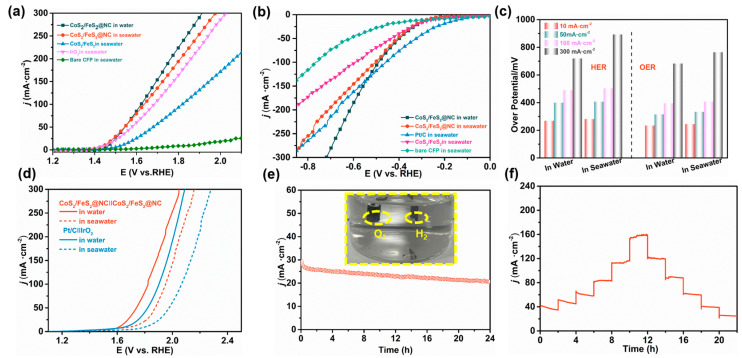
LSV curves for (**a**) OER and (**b**) HER in alkaline seawater; (**c**) comparation of overpotentials with CoS_2_/FeS_2_@NC to deliver 10, 50, 100, and 300 mA·cm^−2^ in alkaline water or seawater; (**d**) polarization curve of Pt/C||IrO_2_ and CoS_2_/FeS_2_@NC||CoS_2_/FeS_2_@NC in 1.0 M KOH seawater with a scan rate of 5 mV·s^−1^ for overall water splitting; (**e**) i−t curves of CoS_2_/FeS_2_@NC||CoS_2_/FeS_2_@NC at 25 mA·cm^−2^ for overall seawater splitting (insert shows a digital photograph showing the evolution of H_2_ and O_2_ gas from the electrodes); (**f**) multistep chronopotentiometry of CoS_2_/FeS_2_@NC||CoS_2_/FeS_2_@NC.

As the most abundant water resource on this planet, electrochemical seawater splitting is greatly beneficial to the coastal countries and islands where fresh water is scarce, which can be easily combined with renewable power-generation technologies related to the ocean (such as wave, solar, and wind energy) [40]. However, electrocatalytic seawater splitting is extremely challenging due to its highly corrosive nature, resulting in activity and stability issues impacting most of the catalyst materials examined. Therefore, the search for an active and durable HER catalyst that can be used in seawater is of great significance. In this work, the catalytic activity of CoS_2_/FeS_2_@NC was also further evaluated in alkaline seawater to investigate the universality of the as-prepared electrocatalyst. The seawater obtained from Jiaozhou Beach (Qingdao, China) was alkalized with KOH to reach a measured pH value of ~14, and the precipitations and small particulates in alkaline seawater were removed by centrifugation before the electrocatalytic processing. As shown in Figure 6a, CoS_2_/FeS_2_@NC had superior electrocatalytic activity of OER with the smallest overpotential of 460 mV to deliver 100 mA cm^−2^ relative to IrO_2_ (600 mV) and the CoS_2_/FeS_2_ (660 mV). Particularly, within all the applied potentials, the catalytic activity of CoS_2_/FeS_2_@NC surpassed that of the Ir/O_2_ benchmark under alkaline seawater conditions, demonstrating the enormous potential of the as-designed electrocatalyst for application in seawater. Figure 6b exhibits the HER LSV curves obtained from CoS_2_/FeS_2_@NC, Pt/C, CoS_2_/FeS_2_, and bare CFP electrodes in alkaline seawater. Although the HER activity of CoS_2_/FeS_2_@NC was slightly lower than that of commercial Pt/C, the enhanced catalytic activity was obviously achieved on CoS_2_/FeS_2_@NC relative to the CoS_2_/FeS_2_, showing that the carbon coating synthesis strategy could improve the catalytic activity of the catalyst for HER in seawater. These overpotentials of CoS_2_/FeS_2_@NC for achieving j of 10, 50, 100, and 300 mA cm^−2^ in alkaline water/seawater conditions for OER and HER are summarized in Figure 6c.

Based on the above analyses, an alkaline electrolyzer was self-made by using CoS_2_/FeS_2_@NC as both the anode and cathode for overall water/seawater splitting. The commercial Pt/C||IrO_2_ benchmarks were also applied to assemble the electrolyzer for comparison. Electrochemical tests shown in Figure 6d revealed that the CoS_2_/FeS_2_@NC||CoS_2_/FeS_2_@NC water electrolyzer needed a voltage of 1.67 V to drive 100 mA·cm^−2^, which was lower than that of the noble metal-based IrO_2_ || Pt/C (1.75 V) and also superior to that of many other self-supported bifunctional electrocatalysts (Table S1, Supplementary Information). For overall seawater splitting, CoS_2_/FeS_2_@NC||CoS_2_/FeS_2_@NC required voltages of 1.80 V to drive current densities of 100 mA cm^−2^ in a 1 M KOH seawater solution (Figure 6d), which was better than the Pt/C||IrO_2_ couple and other similar electrocatalysts (Table S2, Supplementary Information). Additionally, the gap between the overall water and seawater splitting performance of the CoS_2_/FeS_2_@NC||CoS_2_/FeS_2_@NC was much narrower than that of the Pt/C||IrO_2_ benchmark (Table S3, Supplementary Information), confirming that the electrocatalytic activity was well maintained in seawater, matching well with the LSV curves of OER and HER. Finally, the durability of this electrolyzer in water and seawater was examined by the long-term i-t curves, and the results showed that CoS_2_/FeS_2_@NC||CoS_2_/FeS_2_@NC has excellent electrocatalysis stability at the current density of 25 mA cm^−2^ for water splitting with the operation time up to 24 h (Figure S7). For overall seawater splitting, the i-t curve slightly decreased (16% after 24 h), which may have been due to the weak adhesion of the catalyst on the surface of the CFP paper, resulting in some catalyst falling off during the catalytic process (Figure 6e). From the inserted digital photograph in Figure 6e, it can be obviously seen that abundant bubbles were generated on the electrodes owing to the low overall seawater splitting potentials (Movie S1, Supplementary Information). Moreover, CoS_2_/FeS_2_@NC||CoS_2_/FeS_2_@NC also displayed remarkable stability with negligible changes in current densities and voltage attenuation via the step chronopotentiometry measurements (Figure 6f). The discussion demonstrated that the core–shell CoS_2_/FeS_2_ heterojunction provides tremendous potential as an excellent and stable electrocatalyst for bifunctional water/seawater splitting.

## 4. Conclusions

In summary, the core–shell CoS_2_/FeS_2_ heterojunctions encapsulated in N-doped carbon nanocubes were prepared by simple synchronous carbonization and sulphuration with the help of dopamine self-polymerizing on the surface of the coordination polymer precursor, which was applied as a bi-functional electrode material for electrocatalytic alkaline water/seawater splitting. The heterogeneous combination of CoS_2_ and FeS_2_ could provide multi-interfaces to optimize the active sites to improve the electrocatalytic activity, and the unique morphology of core–shell carbon nanotubes not only increases the quantity of accessible active sites but also enhances the stability during catalytic processing. As expected, CoS_2_/FeS_2_@NC served as both a cathode and anode material and could drive the water splitting in alkaline media with 1.67 V at the current density of 100 mA cm^−2^, and it maintained long-term stability at large current densities, which was better than the commercial Pt/C and IrO_2_ electrolyzer. For alkaline seawater splitting, the CoS_2_/FeS_2_@NC||CoS_2_/FeS_2_@NC combined system also demonstrated satisfactory overpotential (1.80 V at 100 mA cm^−2^) and great durability in seawater electrolytes. This study may open up avenues to design bimetallic water/seawater splitting electrocatalysts with bi-functional features.

## Data Availability

Data are contained within the article or Supplementary Materials.

## Supplementary Materials

The following supporting information can be downloaded at: https://www.mdpi.com/article/10.3390/polym17121701/s1, Figure S1: SEM images of CoFe-CPs (a,b) and (c,d) CoFe-CPs@PDA; Figure S2: N2 adsorption-desorption isotherm of CoS2/FeS2@NC at 77 K; Figure S3: XRD patterns of CoFe-CPs (a) and (b) CoS2/FeS2; Figure S4: OER (a) and HER (b)curves of CoS2/FeS2@NC obtaining at different anealing temperature; Figure S5: OER (a) and HER (b)curves of CoS2/FeS2@NC obtaining with different ratio of CoFe-CPs@PDA and dopamine; Figure S6: Cyclic voltammogram (CV) curves of (a) CoS2/FeS2@NC, (b) CoFe-CPs@PDA, and (c) CoS2/FeS2; (d) capacitive currents as a function of scan rate; Figure S7: i-t curves of CoS2/FeS2@NC||CoS2/FeS2@NC at 25 mA·cm^−2^ for overall water splitting; Table S1: Overall water splitting performance comparison of CoS2/FeS2@NC in 1 M KOH with other bifunctional electrocatalysts reported presently. V100 and V200 represent the cell voltages required for the catalysts to reach 100 and 200 mA cm^−2^, respectively; Table S2: Overall water splitting performance comparison of CoS2/FeS2@NC in 1 M KOH seawater with other bifunctional electrocatalysts reported presently. V100 and V200 represent the cell voltages required for the catalysts to reach 100 and 200 mA cm^−2^, respectively; Table S3: Comparation of voltages gap with CoS2/FeS2@NC and Pt/C|| IrO2 to deliver 100 mA·cm^−2^ in alkaline water or seawater. Video S1: Overall seawater splitting.
