# Supplementary material for: Core–Shell CoS2/FeS2 Heterojunction Encapsulated in N-Doped Carbon Nanocubes Derived from Coordination Polymers for Electrocatalytic Alkaline Water/Seawater Splitting
Source: Polymers (Basel). 2025 Jun 19;17(12):1701. doi: 10.3390/polym17121701 (PMC12196570; doi:10.3390/polym17121701)
Supplement: Supplementary file 1 [file polymers-17-01701-s001.zip › Supplementary Information.pdf]

Core–Shell CoS_2/FeS_2 Heterojunction Encapsulated in N-Doped Carbon Nanocubes Derived from Coordination Polymers for Electrocatalytic Alkaline Water/Seawater Splitting

Xiaoyin Zhang [1], Yan Liu [1], Zihan Zeng [1], Yan Zou [1], Wanzhen Wang [2], Jing Zhang [1], Jing Wang [3,*], Xiangfeng Kong [1,*] and Xiangmin Meng [2]

1 Institute of Oceanographic Instrumentation, Qilu University of Technology (Shandong Academy of Sciences), Qingdao 266061, China
2 College of Marine Science and Biological Engineering, Qingdao University of Science and Technology, Qingdao 266042, China
3 Modern Industrial College of Biomedicine and Great Health, Youjiang Medical University for Nationalities, Baise 533000, China
* Correspondence: wangjing@ymun.edu.cn (J.W.); kxf_1985@163.com (X.K.)

Experimental section

Materials

All chemicals, including cobalt (II) nitrate hexahydrate ($CoNO_3{\cdot}6H_2O$, AR), potassium hexacyanoferrate (III) ($K_3[Fe(CN)_6]$, AR), trisodium citrate dihydrate ($Na_3C_6H_5O_7{\cdot}2H_2O$, AR), dopamine (AR), sublimed sulfur (S, AR), ethanol (AR), and Nafon (5 wt %) were purchased from Sigma-Aldrich and used as received without any purification. Deionized water (DI, 18 MΩ) used for all experiments was supplied by a Millipore system (Millipore Q).

Synthesis of Co-Fe hybrid coordination polymer (CoFe-CPs)

CoFe-CPs was synthesized through a coprecipitation method, $CoNO_3{\cdot}6H_2O$ (12 mmol, 3.49 g) and $Na_3C_6H_5O_7{\cdot}2H_2O$ (15mmol, 4.42 g) were dissolved in 200 mL of DI water to form solution A. $K_3[Fe(CN)_6]$ (8mmol, 2.63 g) was dissolved into 100 mL of DI water to form solution B. Then, solutions A and B were thoroughly mixed under magnetic stirring for 2 h, followed by 22 h aging. The obtained precipitates solid was collected by centrifugation, washed with DI water and ethanol for several times, and then dried overnight at 70 °C in oven.

Synthesis of polydopamine coated CoNi-CPs hollow cubes (CoFe-CPs@PDA)

0.2 g of CoFe-CPs nanocubes were dispersed into 100 mL Tris-buffer solution (pH = 8.5,10 mM) with ultrasonication for 20 min, and then 0.04 g dopamine was added into solution, followed by stirring for 24 h. The resultant was collected *via* centrifugation and washed with deionized water and ethanol for several times, respectively. The final product was dried under vacuum at

50 °C overnight. In addition, the other four samples with different addition of dopamine (0.02, 0.05g, 0.1g and 0.2g) were prepared to investigate the effect of PDA loading amounts.

Synthesis of CoS_2/FeS_2@NC nanocubes

In a typical procedure, the porcelain boat with the prepared CoFe-CPs@PDA precursor (100 mg) were placed at the center of the tube furnace, with 300 mg of sulfur powder placed at the upstream side of the furnace as the S source. Impurity gas in the furnace was purged with Ar-gas (purity, 99.999%) for 30 min. Afterward, the furnace was the tube was heated to 400°C with a rate of 2 °C min^{-1} under the flowing Ar-atmosphere and kept at this temperature for 3 h. Finally, the furnace was naturally cooled down to room temperature with Ar-gas flowing. The confined biactive CoS_2/FeS_2 into N-doped carbon nanocubes (CoS_2/FeS_2@NC) were obtained. The effect of pyrolysis temperatures (300 and 500 °C) was also explored, the products were denoted as CoS_2/FeS_2@NC-300 and CoS_2/FeS_2@NC-500, respectively. Additionally, CoS_2/FeS_2 was prepared using the same condition with CoS_2 /FeS_2@NC excepting that CoFe-CPs@PDA was replaced with CoFe-CPs.

Characterization

The morphologies of as-prepared samples were analyzed by a field-emission scanning electron microscope (SEM, S-4800, Hitachi), transmission electron microscopy (TEM, Tecnai G2-F20), high-resolution transmission electron microscopy (HR-TEM, JEOL JEM-2100F) and high-angle annular dark-field scanning transmission electron microscope (HAADF-STEM, JEM-ARM200F, 200 kV). The crystal structure was examined by X-ray diffraction (XRD, Bruker D8-Advance diffractometer) using Cu Kα radiation. The valence states were detected by X-ray photoelectron spectroscopy (XPS, AXISULTRA DLD) on a Phi X-tool XPS instrument with Al Kα X-ray source. Raman spectra was performed on a laser Raman spectrometer (Renishaw in via plus) with an excitation laser of 514 nm. The obtained adsorption-desorption isotherms were evaluated on a Micromeritics Tristar II 3020M to give the pore parameters including Brunauer-Emmett-Teller (BET) specific surface area and pore size. The pore size distribution was calculated from the HK method.

Electrocatalytic measurements

In a typical prepared procedure of the working electrode, 50 μL of the homogeneous ink, which was prepared by dispersing 8 mg sample and 80 μL Nafion solution (5 wt%) in 1920 μL ethanol solution, was loaded onto the two sides of the carbon fiber paper (CFP) electrode (0.5×1 cm^2) with the desired loading mass of 0.4 mg/cm^2. Prior to use, the CFP (FuelCell Store) was treated

in a mixed solution of sulfuric acid and nitric acid (v, 98% H_2SO_4): v (70%, HNO_3): v(H_2O) = 1:1:1 at 60 °C for 24 h under vigorous magnetic stirring.
All electrochemical measurements were conducted at room temperature (≈25 °C) in a typical three electrode or two-electrode configuration using a CHI 760 E Electrochemical Workstation (CHI Instruments, Shanghai Chenhua Instrument Corp., China). The HER performance was evaluated in alkaline seawater (pH = 14) or 1.0 M KOH (pH = 14) solution with as-fabricated CFP as the working electrode, a Ag/AgCl as the reference electrode, and graphite rod as the counter electrode. The alkaline seawater was

The OER performance was evaluated with the same three-electrode in 1.0 M KOH solution or alkaline seawater except that a Pt plate was used as the counter electrode. The full electrolyzer cell was assembled using two identical CFP electrodes with as-fabricated CoS_2 /FeS_2@NC and measured in a two-electrode mode. In this report, all potentials were scaled with respect to the RHE by the following equation: $E_{RHE} = E_{Ag/AgCl} + 0.197 + 0.059 \times pH$. The linear sweep voltammetry (LSV) curves were collected with a scan rate of 5 $mV \cdot s^{-1}$. Electrochemical impedance spectroscopy (EIS) was tested at a constant potential of 1.60 V (vs. RHE) from 10000 to 0.1 Hz with an AC potential magnitude of 10 mV. The electrical double layer capacitor (C_{dl}) were obtained from CV plots in a non-Faradaic small window of 0.06-0.18 V (vs. RHE). The electrode durability was conducted at 10 $mA \cdot cm^{-2}$ for 36000 s by using chronoamperometry.

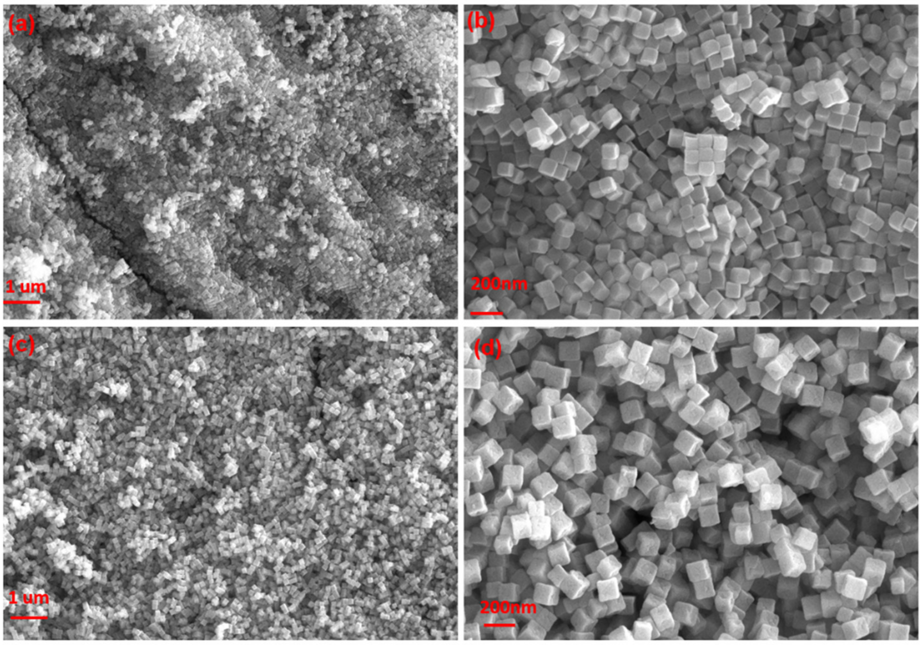

Figure S1. SEM images of CoFe-CPs(a,b) and (c,d) CoFe-CPs@PDA

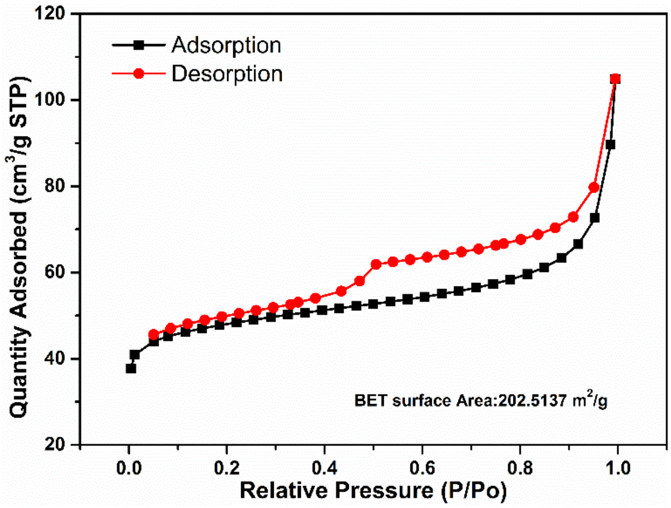

Figure S2. N_2 adsorption-desorption isotherm of CoS_2/FeS_2@NC at 77 K.

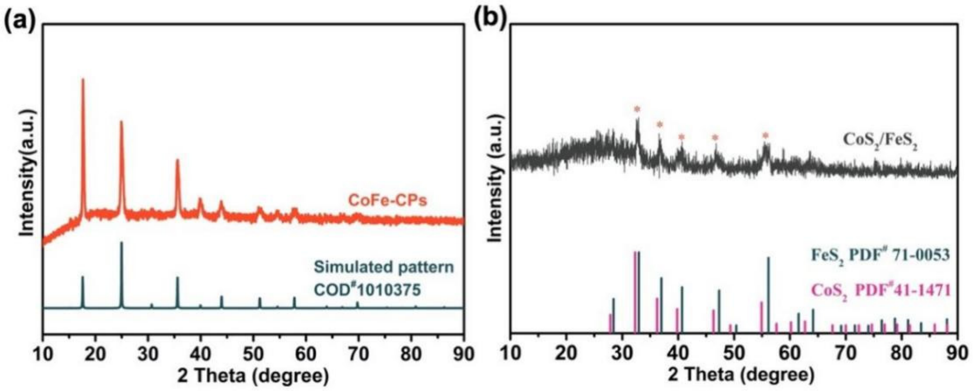

Figure S3. XRD patterns of CoFe-CPs (a) and (b) CoS_2/FeS_2.

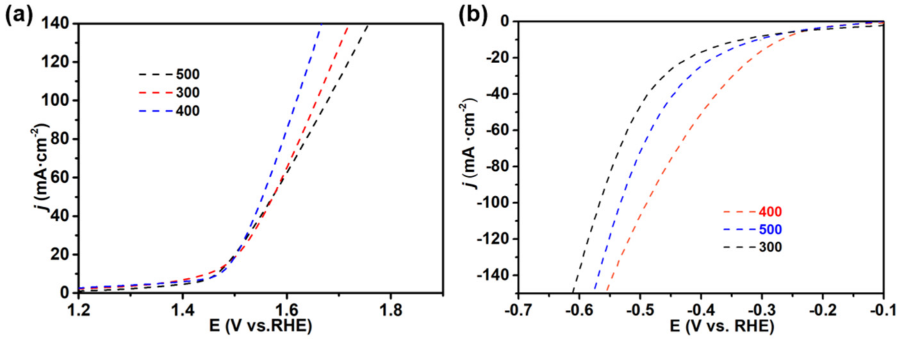

Figure S4. OER(a) and HER (b)curves of CoS_2/FeS_2@NC obtaining at different anealing temperature.

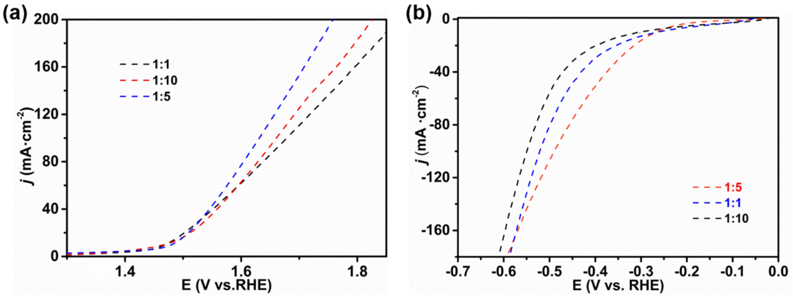

Figure S5. OER(a) and HER (b)curves of CoS_2/FeS_2@NC obtaining with different ratio of CoFe-CPs@PDA and dopamine.

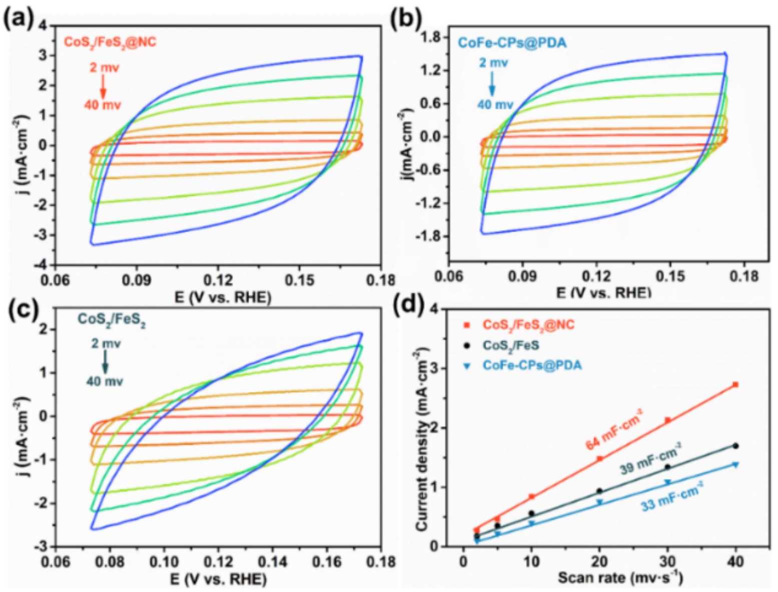

Figure S6. Cyclic voltammogram (CV) curves of (a) CoS_2/FeS_2@NC, (b) CoFe-CPs@PDA, and (c) CoS_2/FeS_2; (d) capacitive currents as a function of scan rate.

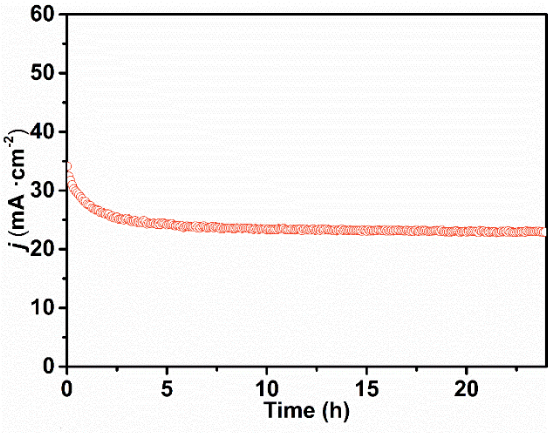

Figure S7. i -t curves of CoS_2/FeS_2@NC||CoS_2/FeS_2@NC at 25 mA·cm^{-2} for overall water splitting.

Table S1. Overall water splitting performance comparison of CoS_2/FeS_2@NC in 1 M KOH with other bifunctional electrocatalysts reported presently. V100 and V200 represent the cell voltages required for the catalysts to reach 100 and 200 mA cm-2, respectively.

Electrocatalyst	V_{100} (V)	References
**CoS_2/FeS_2@NC**	**1.67**	**This work**
**Pt/C‖ IrO_2**	**1.75**	**This work**
$(Ni_{0.33}Fe_{0.67})_2P$/NF	1.72	Adv. Funct. Mater. 2017, 27, 1702513
$Fe_{0.09}Co_{0.13}NiS_2$	1.69	Adv. Mater. 2018, 30, 1802121
$CoSn_2$	1.69	Angew Chem Int Ed 2018, 57, 15237-1524
NiFeRu LDH	1.705	Adv. Mater. 2018, 30, 1706279
FeCoNi-HNTAs	1.725	Nat Commun 2018, 9 (1), 2452
Ni-ZIF/Ni-B	1.78	Adv. Energy Mater. 2020, 10, 1902714
Mo-Ni3S2/NixPy	1.80	Adv. Energy Mater. 2020, 1903891
Ni_2P-Fe_2P	1.682	*Adv. Funct. Mater.* **2021**, *31*, 2006484
CoFe-Ni_2P	1.725	*Adv. Energy Mater.* **2023**, *13*, 2301475

Table S2. Overall water splitting performance comparison of CoS_2/FeS_2@NC in 1 M KOH seawater with other bifunctional electrocatalysts reported presently. V_{100} and V_{200} represent the cell voltages required for the catalysts to reach 100 and 200 mA cm^{-2}, respectively.

Electrocatalyst	V_{100} (V)	References
**CoS_2/FeS_2@NC**	**1.79**	**This work**
**Pt/C‖ IrO_2**	**1.90**	**This work**
Ni_2P-Fe_2P	1.811	*Adv. Funct. Mater.* **2021**, *31*, 2006484
Cr_2O_3-CoO_x	1.81	*Nat. Energy* **2023**, *8*, 264
NixFeyN@C	1.910	J. Mater. Chem. A, 135692021,9, 13562-
Ru-CoOx/NF	1.860	Small 2021, 17, 2102777
CoFe-Ni_2P	1.738	*Adv. Energy Mater.* **2023**, *13*, 2301475
Ru-Ni(Fe)P_2/NF	1.82	*Small* **2023**, *19*, 2300030

Table S3. Comparation of voltages gap with CoS_2/FeS_2@NC and Pt/C‖ IrO_2 to deliver 100 mA·cm^{-2} in alkaline water or seawater

**100mV**	**Water splitting**	**Seawater splitting**	**gap**
**CoS_2/FeS_2@NC**	**1.67**	**1.80**	**0.13**
**Pt/C‖ IrO_2**	**1.75**	**1.90**	**0.15**
